# Phenological Growth Stages of *Buddleja saligna* Willd. According to the BBCH Scale

**DOI:** 10.3390/plants13243542

**Published:** 2024-12-19

**Authors:** Maboka M. Mabusela, Babalwa Matsiliza-Mlathi, Riana Kleynhans

**Affiliations:** Department of Horticulture, Tshwane University of Technology, Private Bag X680, Pretoria 0001, South Africa; matsiliza-mlathib@tut.ac.za (B.M.-M.); kleynhansr@tut.ac.za (R.K.)

**Keywords:** BBCH, phenological growth stages, *Buddleja saligna*, flowering phenology, plant development

## Abstract

*Buddleja saligna* Willd. is an evergreen tree native to South Africa. Historically, the tree has been used for the treatment of various diseases and has been scientifically found to have promising pharmacological effects. In the current study, the phenological growth stages of *B. saligna* are characterised according to the Biologische Bundesanstalt, Bundessortenamt und Chemische Industrie (BBCH) scale standardised coding. A total of eight out of ten principal growth stages have been described for the development of buds, leaves and shoots, inflorescence emergence, flowering, the development and ripening of fruits, and senescence. A total of thirty-three secondary growth stages were identified and described in detail. Illustrative images with codification have been provided to better define and describe the growth stages. The temperature fluctuations during the study period could have influenced the flowering time, as phenological shifts were observed during the study period. These data are a useful reference for efficient management, cultivation, and scientific research.

## 1. Introduction

*Buddleja saligna* Willd. is one of 150 species of the genus *Buddleja*, which belongs to the Scrophulariaceae family. It is a small- to medium-sized evergreen tree commonly known as false olive (English), Witolien (Afrikaans), umBatacwepe (Siswati), Lelothwane (Southern Sotho), Mothlware (Tswana), unGqeba (Xhosa), and iGqeba-elimhlope (Zulu) [1]. It can grow up to 12 m tall as a tree or a multi-stemmed shrub with a 38 cm wide trunk [2,3,4]. The species has the widest distribution in South Africa as it is found in all nine provinces of the country [5,6]. In addition, the species is also found in the eastern highlands of Zimbabwe and is marginal in Swaziland.

Several *Buddleja* species are used in traditional herbal medicine in many parts of the world and have promising pharmacological activities [7,8,9,10,11,12]. *Buddleja saligna* has long been used to treat colds and coughs, diabetes and tuberculosis, irritated eyes, chest pain, wound healing, urinary problems, and to control blood pressure [2,7,10,13,14]. The species has also been reported to have pharmacological properties such as antioxidant, anti-inflammatory, antibacterial, antimutagenic, and anticancer properties [2,6,7,13,15]. Recently, this plant has been reported to possess photoprotective effects and antiproliferative activity against human dermal fibroblasts (MRHF) and human malignant melanoma (UCT MEL 1) and could be considered a useful and viable additive for sunscreen formulations due to its diverse biological activity [6]. To commercialise products from *B. saligna* plant material, a good source of homogeneous quality material is required, which is why it is important to understand and encode the phenological events for agronomic management.

Phenology is the study of the life cycle of plants, especially their timing (often in relation to critical events), which is determined by changes in weather and climate [16,17,18,19]. The description of the developmental stages of plants has been of interest for centuries and was recorded as early as 750 A.D. [20]. In ancient times, farmers observed the growth of plants after sowing and carried out harvests year after year and quickly became aware of the connection between the changes in their environment and the development of plants [21].

Phenological data are of great economic importance as they provide valuable information on the developmental stages of plants, which is crucial for agronomic management practices [16,17,22,23,24,25]. They also determine the adaptability of plants to different environmental factors and the ability of species to establish under favourable environmental conditions and avoid unfavourable environmental conditions, thus influencing their distribution [26,27,28]. In addition, farmers can determine the timing of sowing, pruning, pesticide application, and frost protection, and thus predict the time of ripening, yield, and quality of crops, which improves market supply [29,30,31,32]. Phenological data can also help to determine the best time to harvest medicinal plants, as the secondary metabolites change mainly with the morphological and phenological stages of the plants as well as the environmental conditions [17,29,33,34]. Determining the optimum harvest time is crucial to ensure that the plants achieve their intended cultivation goal.

Developmental stage systems can therefore be useful tools for standardising communication among those involved in agricultural activities, such as farmers, extension workers, crop insurers, educators, and scientists, and they are a tool for providing agricultural practises [35,36,37]. Until the early 1990s, there was no standardised coding method for describing phenological growth stages [38]. Most of these descriptions were based on the Fleckinger scale, which uses a combination of letters and numbers and deals exclusively with inflorescence development [16]. The further development of the phenological recording methodology is the BBCH scale (Biologische Bundesantalt, Bundessortenamt und Chemische Industrie), which is characterised by its simplicity and ease of use for plants from annuals to perennials and describes both the vegetative and reproductive phases of plant growth [39].

The BBCH scale is a system for uniformly coding phenologically similar growth stages of all monocotyledonous and dicotyledonous plants. The entire development cycle of plants is divided into ten clearly recognisable and distinguishable, long-lasting developmental stages [40]. These principal growth stages are described with numbers from 0 to 9 in ascending order. In addition, secondary stages are used when points in time or steps in plant development need to be specified precisely. In contrast to the principal growth stages, secondary stages are defined as short developmental steps characteristic of the respective plant species that are passed through in succession during the respective principal growth stages and are also coded with a scale from 0 to 9. The two-digit code is a scale that offers the possibility to precisely define all phenological growth stages for most plant species [40,41].

Recently, an extended BBCH scale has been introduced with a three-digit code for some specific stages of plants. The three-digit code for the mesostage allows a further subdivision of these secondary growth stages [36,42]. The basic BBCH scale and the extended BBCH scale are widely considered reliable for describing the phenological growth stages of horticultural crops, medicinal plants, aromatic plants, etc., as they provide a more detailed and accurate description of the main phenological events, allowing appropriate timing for management practices [32,43,44,45]. The data are also important for ordinary people who collect plant material, as collecting is not only linked to the season because the phenological stages can shift, especially due to climate change. The aim of this study therefore was to investigate the phenological growth stages of *B. saligna* according to the BBCH scale.

## 2. Results

**Figure 1:**
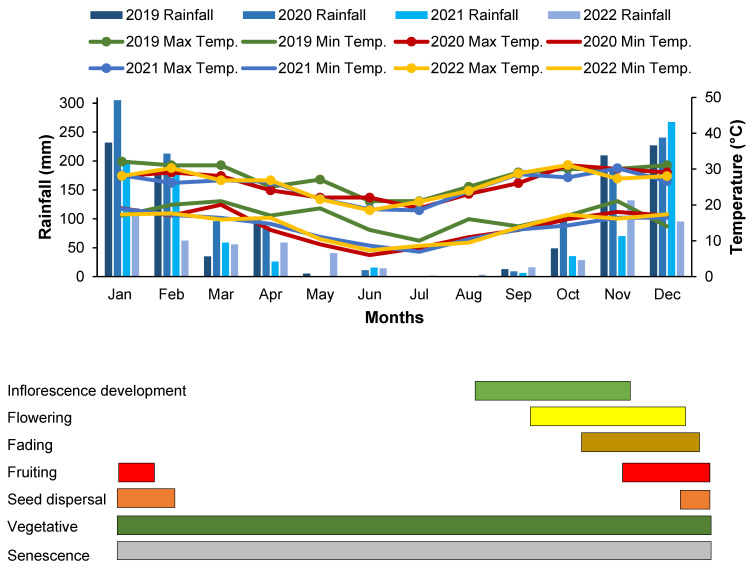
Chronological progression of the principal growth stages of the *Buddleja saligna* scale and weather data of the experimental site in Mamelodi from 2019 to 2022 (weather data accessed from [46]). The vertical bars indicate the amount of rainfall during the study period, while the horizontal bars indicate the time elapsed in each stage.

*Buddleja saligna* grew throughout the year with defined periods of slow growth and growth flush and the phenological growth stages overlapping throughout its life cycle (Figure 1). Although leaf yellowing also occurred all year round, it was at its peak during winter when the growth was at its lowest and decreased in spring when the plants entered the flowering phase. The reproductive phase of *B. saligna* began towards the end of winter and lasted until the end of summer, depending on the weather conditions of the respective year of observation (Figure 1).

Generally, 2019 was warmer than 2020, 2021, and 2022, with higher minimum temperatures than in the other years. In the first five months of 2019, the maximum temperature was higher than in the other years, with the exception of April, while the minimum temperature in the same year was consistently higher than in the other years, with the exception of September and December. On the other hand, in general, 2020 was colder than the other years, as it reached the lowest recorded temperature in June compared to 2019, 2021, and 2022 (Figure 1).

The highest maximum temperatures were between 29 and 31 °C in the summer months, while the minimum temperatures in the winter months dropped below 10 °C in June and July. In Mamelodi, most of the rainfall occurred from late spring (November) to late summer (February), and the winter months were generally dry with less than 50 mm in June and 0 mm in July. The year 2020 was the rainiest year with a higher amount of rainfall and the highest amount of rainfall fell in January with almost 300 mm, while the year 2022 generally had less rainfall (Figure 1).

The phenological study, using BBCH scale coding, covered the entire annual cycle of *B. saligna*. This covered eight of the ten principal growth stages of the BBCH scale (Table 1). The principal growth stages 2 (development of lateral shoots) and 4 (development of harvestable vegetative plant parts) were omitted as they do not apply to *B. saligna*, while principal growth stage 7 (development of fruits) was not described in detail due to the tiny size of *B. saligna* seeds. Although the principal growth stage (dormancy) was taken into account, it was measured by the percentage of yellow leaves (senescence) during the winter period when the tree’s growth was minimal, as *B. saligna* is an evergreen tree and does not fully enter senescence.

### 2.1. Vegetative Phase: Principal Growth Stages 0–3

**Figure 2:**
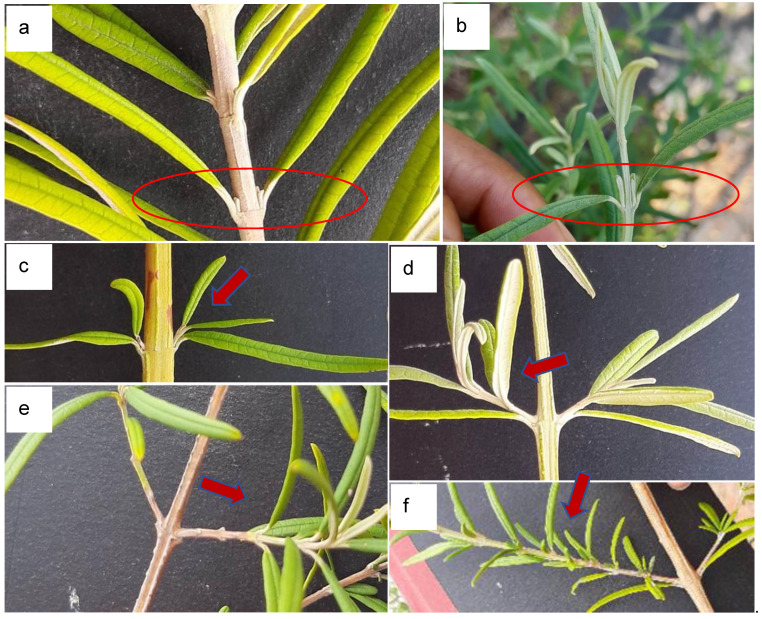
Vegetative growth stages of *Buddleja saligna* according to the BBCH scale: (**a**) bud development (BBCH scale 00); (**b**) bud burst (BBCH scale 09); (**c**) development of new leaves (BBCH scale 11); (**d**) leaves fully developed (BBCH scale 19); (**e**) shoot development (BBCH scale 33); and (**f**) shoot fully developed (BBCH scale 39).

Principal growth stage 0 covers vegetative bud development of *B. saligna* from the time when the buds are dormant, completely closed, and covered with grey–green scales (BBCH scale 00) to the end of bud break/burst (BBCH scale 09) (Figure 2a,b). The higher the second digit, the higher the progression of the principal growth stage, i.e., BBCH scale 01 indicates the beginning of bud swell; BBCH scale 03 indicates the end of bud swell. Although leaf buds occurred throughout the year, most of these buds were more evident in winter, during the period of slow growth, and bud bursts occurred in large numbers from spring to autumn.

Principal growth stage 1 describes leaf development from the separation of the first leaves (BBCH scale 10) to the time when all leaves are fully unfolded and expanded (BBCH scale 19) (Figure 2d). Most of the leaves developed in autumn during the vegetative phase, shortly after the plant stopped the reproductive phase resulting in a growth flush. Principal growth stage 1, leaf development (BBCH scale 10-19), occurred simultaneously with the principal growth stage 3 (shoot development). The pronounced shoot visibility occurred towards the end of leaf development (BBCH scale 19). This process continued until the middle of winter when vegetative flush and the elongation of the leaf tip were clearly visible. The first two leaves unfolded at stage 11 (BBCH scale 11) (Figure 2c) and by stage 19 (BBCH scale 19) all leaves had unfolded and were 90% of their final size (Figure 2d).

Principal growth stage 3 focuses on the development of shoots and the increment of the second digit denotes the progression in the length development (number of nodes) of the shoots from shoot initiation (BBCH scale 31) until it has several nodes (BBCH scale 39). Eventually, the leaves turned dark green, and the shoots became woody within 5–7 weeks. As shoot development progresses, the old leaves eventually fall off (BBCH scale 33) (Figure 2e).

### 2.2. Reproductive Phase

**Figure 3:**
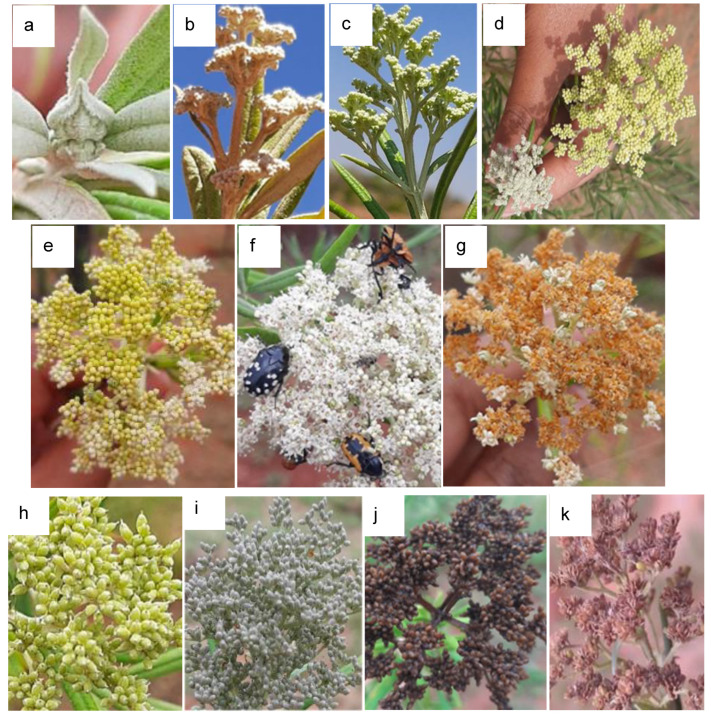
Reproductive growth stages of *Buddleja saligna* according to the BBCH scale: (**a**) enclosed floral bud (BBCH scale 50); (**b**) inflorescence is 50% of the final size (BBCH scale 55); (**c**) inflorescence is 70% of the final size (BBCH scale 57); (**d**) end of inflorescence expansion with a colour change (green to cream) of flower buds (BBCH scale 59); (**e**) beginning of flowering, with 10% of flowers opened (BBCH scale 61); (**f**) full flowering (BBCH scale 65); (**g**) fading stage, with almost all the petals dropped (BBCH scale 69); (**h**) fruits reached their final size but green (BBCH scale 79); (**i**) fruits turned grey (BBCH scale 81); (**j**) fruits turned brown, BBCH scale 85; and (**k**) seeds dispersed, BBCH scale 89.

The reproductive phase comprises four principal growth stages (Figure 3): inflorescence emergence (stage 5), flowering (stage 6), fruit development (stage 7), and fruit ripening (stage 8). This phase was recorded from mid-August, when the first floral buds were observed, to the end of February when the last seeds were dispersed.

#### 2.2.1. Principal Growth Stage 5: Inflorescence Emergence

The principal growth stage 5 qualitatively describes the morphology of the developing inflorescence (Figure 3a–d). The inflorescence is a panicle that usually develops on the aerial parts of the plants. A tree with a crown diameter of ±2 m can produce about 400–500 inflorescences and an inflorescence can bear 350–450 flowers. In addition, 80% of the inflorescences appear in the upper part of the tree crown. This stage usually begins in late winter towards the end of August with the appearance of the flower buds when temperatures begin to rise. The buds were visible when scouting during this period, but 70% of the flower buds appeared in early spring (September) and then declined in mid-December during the summer season. In the beginning, the inflorescence bud develops between the young leaves, which curl up when the bud starts to grow. Inflorescence differentiation (BBCH scale 55) (Figure 3b) started three weeks after bud emergence. It also took five weeks after bud emergence for the flower buds to separate (BBCH scale 57) (Figure 3c), and this stage ends when the inflorescence is fully developed, the colour of the flower buds changes from green to cream, and the first petals of the first flowers are visible outside the sepals (BBCH scale 59) (Figure 3d).

#### 2.2.2. Principal Growth Stage 6: Flowering

Flowering takes place when BBCH scale 60 is reached, which is when the first flowers open. The subsequent stages of principal stage 6 indicate the percentage of open flowers on a main shoot, i.e., BBCH scale 61 means that only 10% of the flowers are open (Figure 3e). An inflorescence took about six weeks to fully open flowers (BBCH scale 65) (Figure 3f) after the flower buds had emerged (BBCH scale 50) (Figure 3a), and an additional two weeks (8 weeks from bud emergence) for the flowers to dry out (fade; BBCH scale 69) (Figure 3g). During full flowering (BBCH scale 65), pollinators such as bugs, bees, flies, and beetles were observed in large numbers on the flowers (Figure 3f).

**Figure 4:**
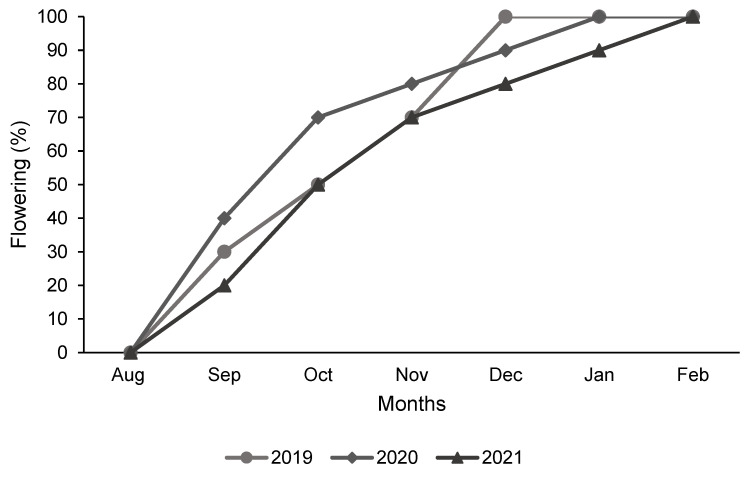
Cumulative flowering percentage (phenological growth stage 6) of *Buddleja saligna* trees from August to February for three different years of phenological observations.

The length of the flowering period of *B. saligna* depends strongly on environmental factors such as temperature and precipitation. Phenological shifts in response to environmental changes were more pronounced in the reproductive phase (Figure 4). In 2019, the reproductive phase started in the first week of August (winter) and lasted until the end of January of the following year. In 2021, the reproductive phase started in late winter, at the end of August, two weeks later than in 2020, and lasted until the end of February/beginning of March, which was longer compared to the previous year (2019). In 2020, the reproductive phase began in mid-August and was completed by mid-February. The flowering phase (principal stage 6) started earlier in 2020 and 70% of inflorescences were open in October, while in 2019 and 2021, only 50% of flowers were open in the same month. In 2021, the trees only reached 100% flowering in February of the following year, which is two months later than in 2019 and one month later than in 2020.

#### 2.2.3. Principal Growth Stage 7: Fruit Development/Growth

The principal growth stage 7 (fruit development) was difficult to document and only two secondary stages were identified: BBCH scale 70 (beginning of ovary growth) and BBCH scale 79 (Figure 3h), with 90% of the final fruit size reached, as the fruits of *B. saligna* are tiny (2 mm) and it is difficult to visually recognise size increments. After the flowers shed their petals, a pistil with a black stigma can be seen, as the ovary develops into a fruit.

#### 2.2.4. Principal Growth Stage 8: Maturity/Ripening of Fruit

This phenological stage is characterised by the colour changes in the fruits from green to brown until they disperse their seeds (BBCH scale 89). The fruits ripen in December and turn from green (BBCH scale 79) to grey (BBCH scale 81) and then brown (BBCH scale 85) before they can disperse seeds (BBCH scale 89) (Figure 3h–k). It took nine to ten weeks from bud burst to fruit ripening. *Buddleja saligna* produces dehiscent fruits that burst open to disperse the seeds, and the inflorescences do not detach after flowering as they can still be seen in the next flowering season. Fruit ripening takes up to a month, from fruit development to seed dispersal.

### 2.3. Senescence

#### Principal Growth Stage 9: Senescence and Beginning of Dormancy

*Buddleja saligna* is an evergreen tree and does not lose all its leaves in winter, although the number of yellow leaves peaks at this time. Phenological growth stage 9 was taken into account, but only the number of yellow leaves was quantified as a percentage. Although the tree did not go into complete winter dormancy, growth was minimal and there were several dormant buds. The number of yellow leaves peaked in winter when about 30% of the leaves (BBCH scale 93) were yellow. However, during the rainy season in summer and during the growth flush that occurred immediately after the reproduction phase, the number of yellow leaves was minimal to almost non-existent.

## 3. Discussion

The description and identification of different phenological stages of a plant species are necessary for planning cultivation practises, characterising germplasm, crop improvement programmes, and studying the impact of climate change on crop production [47,48]. To effectively monitor phenological development and make comparable observations, it is essential to accurately determine the phenological growth stages for each plant species [49] by using a standardised coding like a BBCH scale. In the present study, the different phenological stages of *B. saligna* were identified and described for the first time using the BBCH scale. A total of eight principal growth stages and 33 secondary growth stages were identified, including both vegetative and reproductive growth stages. The number of principal growth stages identified in false olive is similar to most evergreen trees such as *Mangifera indica* L. [38], *Aegle marmelos* [47], *Persea americana* [50], and *Anacardium occidentale* L. [48]. This demonstrates the flexibility of the BBCH scale to accommodate crop-specific stages within a wide range of annuals, perennials, and woody plants.

Principal growth stages 2 (development of lateral shoots) and 4 (development of harvestable parts) were omitted in the current study as the principal growth stage 2, was limited to the description of the main stem observed after seed germination [51], and older trees were used in the present study. The fourth principal growth stage, on the other hand, is not applicable to woody plants but focuses on plants with harvestable parts such as root vegetables, e.g., carrot (*Daucus carota*), beetroot (*Beta vulgaris*), and sweet potato (*Ipomoea batatas*) [45]. In addition, principal growth stage 7 (fruit development) was not described in detail due to the small size of the fruit produced by *B. saligna*, as it forms an ovoid capsule of about 2 mm in length. The BBCH scale relies on visual observations for coding [50], making it difficult to monitor fruit expansion in tiny fruits.

*Buddleja saligna* grew all year round, with distinct peaks of slow and rapid growth in the colder months and warmer temperatures, respectively. The period of slow growth where the tree lost a large number of leaves was considered a dormant phase (principal growth stage 9), but only the number of yellow leaves was recorded and quantified as a percentage. This would help to determine the possibility of harvesting the remaining leaves during this stage for analysis of biological activity. It is known that cold temperatures have an unfavourable effect on plant growth and that growth is therefore inhibited [52]. The results of the current study are consistent with the findings of [17], who observed rapid growth at warmer temperatures and slow growth at lower temperatures in an evergreen tree, *Leucosidea sericea*.

In the study of plant phenology, the focus is often on temperature, while other climatic changes are ignored, as the biological effects of temperature are often better understood. Systematic shifts in flowering time have been reported in the context of global warming and predicting phenology has become an important task in monitoring climate change [53,54,55]. In the present study, some notable phenological shifts were observed during the three years of data collection. The trees started and finished flowering earlier in 2019 compared to 2020 and 2021, and according to the weather data during the study period, it was warmer in 2019 than in the other years. The reproductive shifts observed over the three years of data collection in the current study may be due to temperature fluctuations, as temperature influences the timing of the phenological transition, as the onset of leafing and flowering occurred earlier in many species in recent years, apparently in response to higher winter and spring temperatures [55,56]. Due to these observed phenological shifts, it is important to identify recognisable phenological growth stages for agronomic management practises rather than relying on seasons, as phenological stages can shift depending on climate fluctuations [17,57]. Linking chemical composition and bioactivity to a recognisable phenological plant phase can lead to more precise planning of agronomic management practices [17,38].

Temperatures on either side of the physiological optimal range extending to points beyond (heat stress) or below (cold stress) at which plant fitness and survival are compromised significantly are defined as critical thresholds of temperature extremes and both temperature extremes and physiological optimum are species-specific within a defined environment [58]. Temperature fluctuations are known to be the most influential factor in phenological changes, as most plant activities are stimulated by favourable warm temperatures and stressed by cold temperatures. Higher temperatures cause certain plants to flower earlier in summer and spring [59]. When temperatures are cold in winter, deciduous forest trees and orchards go through a dormant phase during which the vegetative growth activity is suspended until favourable growth conditions resume in spring [29]. Even if the temperature at a given time has no influence on vegetative growth, it clearly alters sexual development, compromising fruit and seed set. Small temperature fluctuations hinder the sexual development of plants [60].

## 4. Materials and Methods

### 4.1. Study Site, Planting, and Maintenance of Buddleja saligna Trees

This phenological study was conducted at the Mothong African Heritage site (25°41′50.7″ S 28°20′15.2″ E) in Mamelodi, Pretoria. Mamelodi is situated at an average altitude of 1327.72 m above sea level. The area is situated on a mountain, but the land is not sloping and has fairly rocky and sandy soil. The area receives rainfall in summer (November–February) and has dry winters with an annual rainfall of 951.7 mm, an average maximum temperature of 26.3 °C, and an average minimum temperature of 14.6 °C. A total of 60 trees were planted in an open field two metres apart within and between the rows of a plot. They were irrigated once a week and fertilized with Vita grow^TM^ 2:3:2 (16) (Talborne Organics, Bronkhorstspruit, South Africa) once during the study period in early September 2020. This study was conducted over a period of three years and six months, from July 2019 to December 2022, and the trees were seven years old at the start of this study.

### 4.2. Sampling and Phenological Observations

Ten trees were randomly selected for observation in the plot, excluding the trees at the edge of the plot, and marked with white T-type plant labels. Three branches of each tree were randomly marked from top to bottom with self-adhesive plant labels. Observations on the marked branches were made weekly and bi-weekly in winter. The following data were collected on the marked branches: vegetative development and reproductive phase (from flower bud development to seed dispersal). During the flowering period, fifteen inflorescences were randomly selected from the labelled branches to count the number of flowers produced per inflorescence. In addition, the total number of inflorescences produced per tree was also counted and recorded.

### 4.3. BBCH Scale Coding

The stages of development and morphological characteristics were recorded according to the BBCH scale guidelines [50]. The BBCH scale uses a two-digit numerical code, with the first digit (0 to 9) denoting the principal growth stages and the last digit (0 to 9) denoting the secondary growth stages. The principal growth stages correspond to the 10 main developmental stages of the plant, i.e., development of the vegetative bud (0), development of leaves (1), formation of lateral shoots (2), development/elongation of shoots (3), development of harvestable vegetative plant parts (4), inflorescence emergence (5), flowering (6), development of fruits (7), ripening of fruits (8), and senescence and beginning of dormancy (9). Each principal growth stage was subdivided into secondary growth stages that describe the point in time in a main growth stage. The higher the code numbers, the greater the progression within the same principal growth stage.

## 5. Conclusions

*Buddleja saligna* grew throughout the year, although growth peaked in autumn after the reproduction phase and stagnated in winter. The reproductive phase started towards the end of winter and lasted until the end of summer. The phenological growth stages of *B. saligna* were described for the first time using the BBCH scale. The BBCH scale clearly distinguishes between the different vegetative and reproductive stages of *B. saligna*. Eight principal growth stages and a total of 33 secondary stages of *B. saligna* were identified, and phenological shifts were observed, especially during flowering, which could have been influenced by temperature fluctuations during the study period. All this is important for general crop management planning, especially for disease and pest control, physiological disorders and weed control, flowering inhibition, and fertiliser effectiveness. Moreover, farmers can link phenological stages to harvest time to obtain homogeneous quality material for medicinal purposes, instead of harvesting seasonally, where the phenological shift can alter the phytochemistry of the plants. In addition, the phenological description is useful not only to obtain basic information about the plants’ requirements but also to facilitate the exchange of scientific information between farmers growing these trees under different environmental conditions.

## Figures and Tables

**Table 1:** Description of the phenological growth stages of *Buddleja saligna* according to the BBCH scale.

BBCH Code	Description
Principal growth stage 0: bud dormancy	
00	Vegetative buds dormant (buds covered by grey scales)
01	Beginning of bud swell (bud scale intact)
03	End of bud swell
07	Beginning of bud break (buds are completely visible)
09	End of bud break (leaf primordia are visible)
Principal growth stage 1: leaf development	
10	First leaves separated
11	First leaves unfolded
15	More leaves unfolded, 50% of its final size
19	All leaves unfolded, with all leaflets fully expanded to 90% of the final size
Principal growth stage 2: development of lateral shoots (not applicable to *B. saligna*)	
Principal growth stage 3: main stem elongation	
31	Ten percent final shoot extension
33	Thirty percent final shoot extension
39	Maximum shoot length
Principal growth stage 4: development of harvestable parts (not applicable to *B. saligna*)	
Principal growth stage 5: inflorescence development/emergence	
50	Bud is enclosed by the leaves
51	Buds are uncovered and start to swell
52	Panicle axes begin to elongate
55	Inflorescence 50% of final size
57	Inflorescence 70% of final size
59	The first petals are visible outside sepals, but all flowers are still closed
Principal growth stage 6: flowering	
60	First flowers opened
61	10% of the flowers open
65	50% full flowering
67	70% of flowers opened, with early opened flowers dried out
69	Flower fading, with most of the flowers dried out
Principal growth stage 7: fruit development	
70	Beginning of ovary growth; stigmas are black with tiny ovaries
79	Fruits in standard size and still green in colour
Principal growth stage 8: fruit ripening	
80	The first few fruits turned grey
81	The fruits turned dark greyish in colour/beginning of ripening
85	The fruits are brown in colour and fully ripened
87	Few seeds are dispersed
89	90% of seeds have dispersed
Principal growth stage 9: senescence	
90	A few leaves on a tree turned yellow
91	10% of the leaves turned yellow
93	30% of the leaves turned yellow

## Data Availability

The original contributions presented in this study are included in the article. Further inquiries can be directed to the corresponding author.
